# Cannabinoids as a Natural Alternative for the Management of Neuropathic Pain: A Systematic Review of Randomized Placebo-Controlled Trials

**DOI:** 10.7759/cureus.70021

**Published:** 2024-09-23

**Authors:** Driti Reechaye, Anne Laure Annaick Perrine, Yashil Jahajeeah, Fateema Dookhee, Jared Robinson, Indrajit Banerjee

**Affiliations:** 1 Internal Medicine, Sir Seewoosagur Ramgoolam Medical College, Belle Rive, MUS; 2 Surgery, Sir Seewoosagur Ramgoolam Medical College, Belle Rive, MUS; 3 Pharmacology, Sir Seewoosagur Ramgoolam Medical College, Belle Rive, MUS

**Keywords:** cannabinoids, cannabis, chronic pain, evidence-based medicine, medical marijuana, pain management, randomized controlled trials

## Abstract

Dysfunction or damage to the nervous system may develop into and result in a chronic pain condition known as neuropathic pain. Neuropathic pain is defined as the structural and functional alteration of the somatosensory component of the nervous system. The treatment of neuropathic pain is a complex endeavor, which often requires specialist care and intensive drug therapy. Recently, cannabinoids have emerged as an alternative and natural option for the treatment of chronic pain, with tetrahydrocannabinol (THC) and cannabidiol (CBD) being the most extensively studied neuroactive components. The therapeutic potential of cannabis remains largely underexplored, primarily due to its social stigma and the restrictions that are in place on its cultivation. The primary aim of this systematic review was to explore the therapeutic value of cannabinoids in the management of chronic pain and thus achieve an improved quality of life for those patients.

A systematic review of the literature published over the last two decades was performed using the following databases: PubMed, Cochrane Central Register of Controlled Trials (CENTRAL), Turning research into practice (Trip), and Google Scholar. Studies that were completed and published between January 01, 2000 and August 31, 2024, in English language, were extracted and appraised. A combination of keywords and Boolean operators Cannabis OR Chronic Pain OR End of life OR Pain Management AND Drug therapy was employed for data extraction. The Cochrane risk-of-bias tool for randomized trials (RoB 2) was used for risk-of-bias assessment. The initial search resulted in 125282 articles; 86,781 of the articles were identified as duplicates and were removed from the primary analysis, and 38,501 abstracts were thus screened. Abstracts, case studies, reports, editorials, viewpoints, cross-sectional studies, cohort studies, case-control studies, case series, and letters to the editor/correspondence manuscripts (n =38,492) were furthermore excluded. Nine full-text articles were critically assessed and tested against the inclusion and exclusion criteria, and a further four articles were excluded with a total of five placebo-controlled randomized control studies being ultimately included in the final systematic review.

Compared to placebo, cannabinoids provided significant relief from chronic pain (33% vs 15%) as measured by the visual analog scale. The transdermal application of CBD led to a more pronounced reduction in sharp pain, according to the neuropathic pain scale. Minimal to no side effects were recorded, further highlighting the potential benefits of cannabinoids.

## Introduction and background

Two of the most common chronic pain syndromes encountered in medical practice are fibromyalgia and neuropathic pain. Neuropathic pain is defined as the structural and functional alteration of the somatosensory component of the nervous system. The most common causes of neuropathic pain are associated with diabetes, HIV/AIDS, Shingles, multiple sclerosis, spinal nerve compression, drug-induced viz. anticancer drugs (cisplatin, vincristine), and radiation therapy. The malfunction of the neural pathways of the musculoskeletal system predominantly characterizes fibromyalgia. These dysfunctions cause a pathological amplification of the pain sensation from both injurious and non-injurious stimuli [1,2]. Fibromyalgia affects approximately 2% to 8% of the general population [2]. A higher percentage of 3% to 17% of individuals suffer from neuropathic pain [3].

The main presenting complaint in these conditions is “pain” which can be touched or evoked and is described as burning or crushing in character and sometimes paresthetic in nature [1,3]. Pain is undoubtedly one of the prime components of the body’s protective mechanism. However, experiencing pain persistently and spontaneously, without a noxious stimulus, is quite detrimental to an individual's quality of life. This negatively impacts the patient's lifestyle both physically and emotionally. Superadded to this, these conditions add undue strain on both the psychological state of the patients and their families [3-5]. The first-line treatment of these conditions includes non-steroidal anti-inflammatory drugs, gabapentinoids, tricyclic antidepressants, serotonin norepinephrine reuptake inhibitors, and opioids. Nonetheless, a rough estimate of only a third of those patients suffering from chronic pain experience a 50% reduction in their pain using the above-mentioned drugs exclusively. The occurrence of severe adverse effects further decreases the potency of the drug as these patients use lower doses than recommended. Drugs like opioids are mainly feared for drug habit-forming capacity [4-7].

Recently, the drug of interest has been medical cannabis, mainly its two best-studied neuroactive compounds tetrahydrocannabinol (THC) and cannabidiol (CBD). THC is the psychoactive component of marijuana, whereas CBD is most commonly found in hemp and is not hallucinogenic but is used in the treatment and management of pain, anxiety, and Parkinson's disease [8,9]. The paucity of evidence on the full potential of cannabis as a pain-modulating drug has often been attributed to its illegality and studies with questionable results. However, further studies have shown considerable positive pain reductive effects that are noteworthy [5,9].

Cannabinoids can medically be administered through a vast plethora of routes; mainly oromucosal, inhalational, or transdermal and they differ in the degree of analgesia provided. Cannabinoid receptors (CB) are distributed all over the body, not only in tissues but in the nervous system too. While both CB1 and CB2 are activated by THC, CBD largely stimulates CB2. The receptor's activation leads to cannabis-induced analgesia as the body’s cannabinoid system regulates pain and stress-modulating pathways. The pain experienced by patients is subjectively graded using either the virtual analog scale (VAS), the neuropathic pain scale (NPS), or the Fibromyalgia Impact Questionnaire (FIQ). Compared to the placebo, cannabinoids lead to significant reduction in pain. Not only was the pain controlled/ minimized but the side effects perceived by the patients were also considered as mild [5,8-10].

Cannabis-derived drugs in their most effective formulation and dosage can be a major breakthrough in the treatment of chronic pain. It is thus obvious that a greater emphasis should be put on medical cannabis as a treatment option through larger scale clinical trials. To that aim, this systematic review was conducted to explore the therapeutic value of cannabinoids in the management of chronic pain and thus achieve an improved quality of life for those patients.

## Review

Methodology

The current systematic review was conducted based on eligible studies identified from PubMed, Cochrane Central Register of Controlled Trials (CENTRAL), Turning research into practice (Trip database), and Google Scholar. The following combination of medical subject headings (MeSH) terms was used for data extraction: “Cannabinoids” OR “Cannabis” OR “Chronic Pain” OR “End of life” OR “Pain Management” AND “Drug therapy”. The search strategy and total number of articles screened by titles and abstract are shown in Table 1. 

**Table 1:** Search strategy

Databases	MeSH Terms Used in Various Databases	Total Number of Articles Screened (Title and Abstract)
PubMed database	Cannabinoids[Title/Abstract]) OR Cannabis[Title/Abstract] OR Chronic Pain[Title/Abstract] OR End of life[Title/Abstract] OR Pain Management[Title/Abstract] AND Drug therapy[Title/Abstract] Filters: from 2000 - 2024	363
Cochrane Central Register of Controlled Trials (CENTRAL)	Cannabinoids OR Cannabis OR Chronic Pain OR End of life OR Pain Management AND Drug therapy in Title Abstract Keyword; Filters: Trials and custom range 01/01/2000- 31/08/2024	92250
Trip database	Cannabinoids OR cannabis OR chronic pain OR end of life OR pain management AND drug therapy; Filters: Controlled trials and 2000-2024	14,869
Google Scholar	Cannabinoids OR cannabis OR chronic pain OR end of life OR pain management AND drug therapy; Custom range: 2000-2024	17,800
		125282

Inclusion Criteria

This systematic review included randomized clinical trials (RCTs) aiming at the evaluation of the impact of cannabinoids on fibromyalgia and neuropathic pain, irrespective of their etiologies. Research studies that were completed and published between January 01, 2000 and August 31, 2024, in English language, were included in this systematic review. Double-blinded and placebo-controlled clinical trials were included for data synthesis.

Exclusion Criteria 

Non-randomized clinical trials, cohort studies, case-control studies, cross-sectional studies, conference abstracts, editorials, case studies, reports, viewpoints, case series, letters to the editor/correspondence manuscripts, comparative studies, and observational studies were not included in this systematic review. Trials focusing on the use of cannabinoids in chemotherapy were also rejected.

Outcomes

The primary outcome of interest was any pain-relieving effect of cannabinoids. Secondary outcomes comprised all-cause mortality and any major side effects like gastrointestinal bleeding, fatal cardiovascular events, or psychosis.

Data Synthesis

Four independent researchers (DR, ALAP, YJ, and FD) analyzed the titles and abstracts from various databases. Full texts of the RCTs that met eligibility criteria were then reviewed for final inclusion. The table of data synthesis included year, study authors, design, sample size, control, study population, route of administration of drug, dose, study measures, main findings, limitations, and the study outcome. 

Risk-of-Bias Assessment

Cochrane risk-of-bias tool for randomized trials (RoB 2) was used, where domains were evaluated at low, unclear, and high risk of bias. YJ and FD independently made the quality assessment stemming from five domains: bias from the randomization process, due to deviations from the intended intervention, due to missing outcome, bias in the measurement of the outcome, and in the selection of the selected result. Traffic light plots and weighted bar plots were generated from the summary of the risk of bias assessment data using the Robvis visualization tool [11].

Results

**Figure 1:**
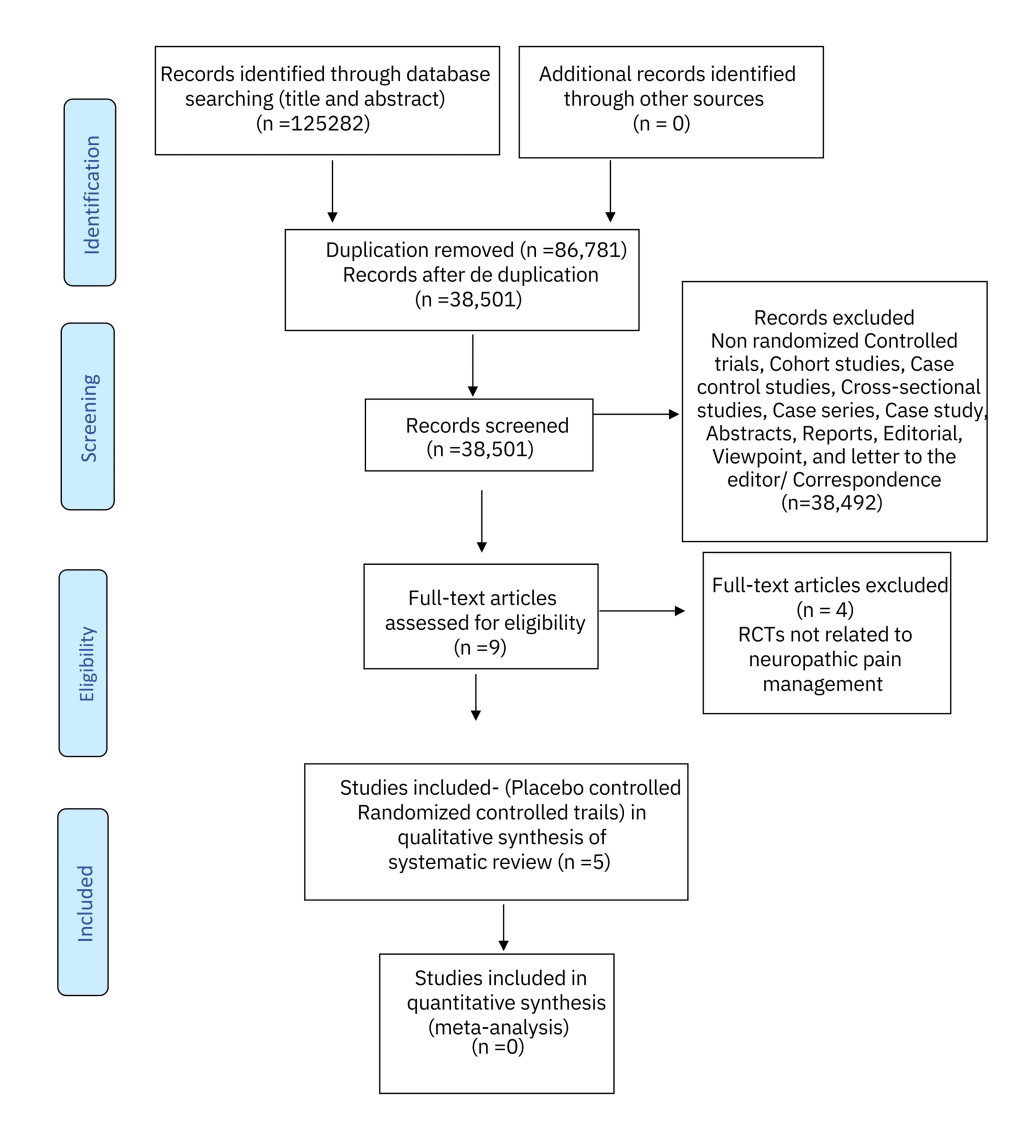
PRISMA flow chart PRISMA: Preferred Reporting Items for Systematic Reviews and Meta-Analyses; RCTs: Randomized Controlled Clinical Trials

The systematic literature search was conducted on four databases viz. PubMed, Cochrane Central Register of Controlled Trials (CENTRAL), Trip database, and Google Scholar for title and abstracts yielded 1,25,282 articles. Out of these, 86,781 were considered as duplicates and excluded from the preliminary analysis. After deduplication, 38,501 manuscripts were left for further screening. The exclusion of non-randomized controlled trials, cohort studies, case-control studies, cross-sectional studies, case studies, case series, reports, abstracts, editorials, viewpoints, and letters to the editor/correspondence manuscripts (n = 38,492) led to a total of nine full-text articles. Taking the inclusion and exclusion criteria into account, the nine articles were then filtered and appraised. Four RCTs were further excluded from the study as their outcomes were not related to neuropathic pain and fibromyalgia management. Ultimately, the five RCTs centered around the efficacy of cannabinoids in the modulation of neuropathic pain and fibromyalgia and they were included in the systematic review (Figure 1).

**Figure 2:**
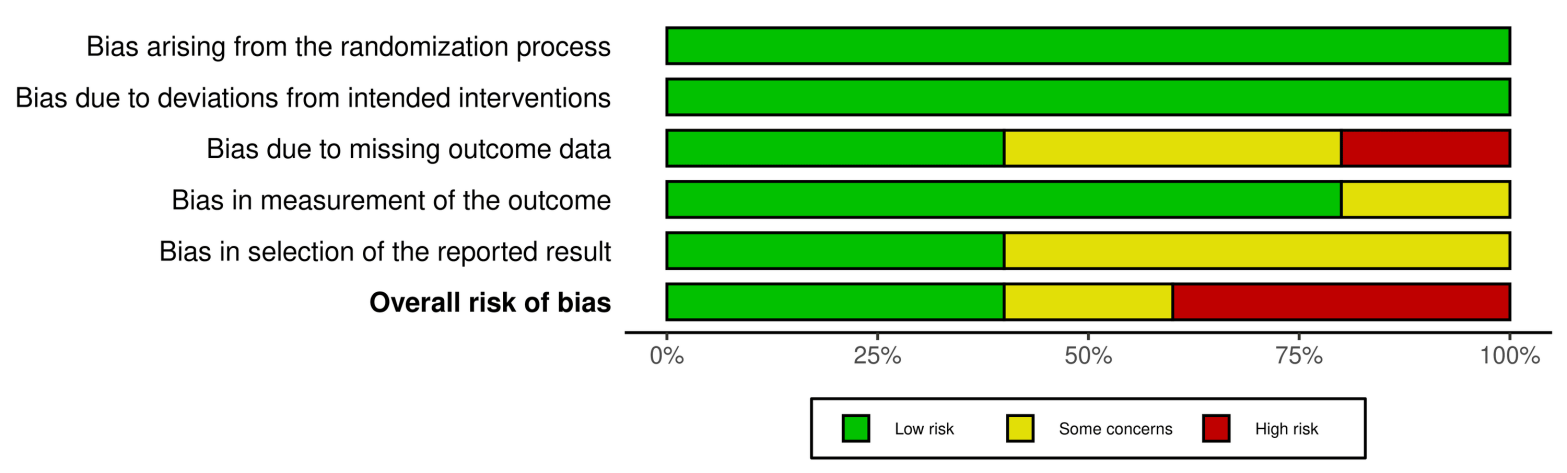
Summary of the risk of bias for RCTs (weighted bar plots) RCTs: Randomized Controlled Clinical Trials

**Figure 3:**
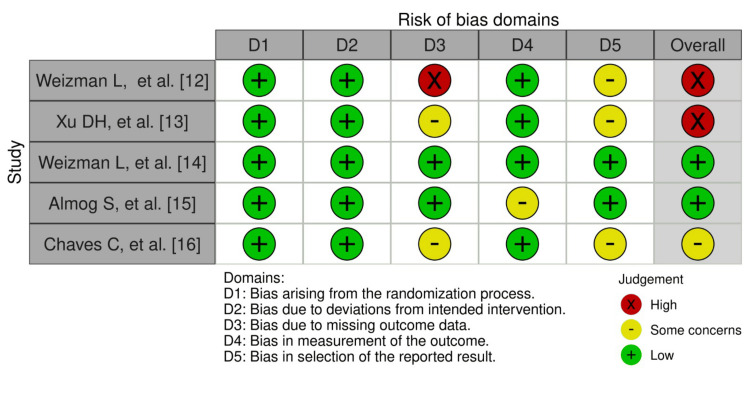
Figure of risk of bias of RCTs (traffic light plot) RCTs: Randomized Controlled Clinical Trials

Figure 2 shows the weighted bar plots for the risk of bias summary and Figure 3 shows a traffic light plot, using the Robvis visualization tool. Quality assessment of the RCTs were analyzed using the RoB 2 tool, which showed overall results of low risk of bias in the randomization process (low risk 100%), deviations from intended interventions (100% low risk), missing outcome data (40% low risk), measurement of the outcome (low risk 80%), and selection of the reported result (low risk 40%), and overall risk of bias for the five RCTs found to be low risk (40%), 20% which signified some concerns and 40% high risk.

Tables 2, 3 depict the country of study, the study design, sample size, the type of intervention, the daily dose, inclusion criteria, the outcomes, the results, and limitations of the interventions of the selected RCTs.

**Table 2:** Country of study, study design, sample size, intervention, daily dose, and inclusion criteria THC: Tetrahydrocannabinol; CBD: Cannabidiol; FM: Fibromyalgia

Author, Year of Study	Country	Study Design	Intervention	Sample Size	Intervention Patients	Control Patients	Daily Dose	Inclusion Criteria
Weizman et al. 2018 [12]	Israel	Randomized Controlled Trial	THC and Placebo	15	9	6	0.2 mg/kg	Neuropathic lower limb radicular pain for more than six months; medium to high chronic pain; no other known comorbidities
Xu et al. 2020 [13]	USA	Randomized Controlled Trial	CBD and Placebo	29	15	14	250 mg/3 fl. oz	Age 18 years or older; both male and female; at least three month-course of symptomatic peripheral neuropathy
Weizman et al. 2024 [14]	Israel	Randomized Controlled Trial	THC and Placebo	15	9	6	0.2mg/kg	Neuropathic lower limb radicular pain for more than six months; medium to high chronic pain; no other known comorbidities
Almog et al. 2020 [15]	Israel	Randomized Controlled Trial	THC and Placebo	27	27	27	0.5 mg 1.0 mg	Age 18 years or older; able to give informed consent; suffering from chronic pain with baseline pain intensity of 6 out of 10 using visual analogue scale
Chaves et al. 2020 [16]	Brazil	Randomized Controlled Trial	THC, CBD and Placebo	18	9	9	THC: 4.4mg CBD: 0.08mg	FM Diagnosis; age 18 years or older; moderate to severe symptoms despite ongoing therapies

**Table 3:** Outcomes, results, and limitations of the selected RCTs HRV: Heart Rate Variability; CPM: Conditioned Pain Modulation; COVAS: Computerized Visual Analog Scale; NPS: Neuropathic Pain Scale; VAS: Visual Analog Scale; NMS:  National Medical Services; FIQ: Fibromyalgia Impact Questionnaire; THC: Tetrahydrocannabinol

Author, Year of Study	Outcome	Results	Limitations
Weizman et al. 2018 [12]	Pain assessment on visual analog scale (VAS); functional brain connectivity	THC-induced analgesia correlates with reduction in functional connectivity between the anterior cingulate cortex and sensorimotor cortex.	Exclusion of women; size of the study population
Xu et al. 2020 [13]	Pain modulation based on neuropathic pain scale (NPS)	Reduced sharp pain; no significant reduction in dull pain	Size of study population; chronic pain experienced from peripheral neuropathy of different etiologies was included in the study.
Weizman et al. 2024 [14]	Assessment of parasympathetic autonomic tone through heart rate variability (HRV) indices; supraspinal pain modulation determined from conditioned pain modulation (CPM) response using computerized visual analog scale (COVAS)	Post-THC administration there is a decrease in HRV indices reflecting a shift toward parasympathetic dominance. This implies reduction in chronic pain which is often associated with sympathetic dominance. Higher reduction in experienced pain through improved CPM response	Exclusion of women; size of the study population; before inclusion in the study, patients were tested for neither cannabinoids nor any other drug abuse
Almog et al. 2020 [15]	Assessment of pharmacokinetics of the drug by doing cannabinoid analysis of the blood samples at NMS Labs; measurement of the pain intensity using VAS.	There was a marked decrease in the VAS score for pain with 1.0 mg of THC as compared to 0.5 mg and placebo. Pain was also noted to decrease more with 0.5 mg of THC as compared to placebo. A notable decrease in pain was observed from 15 minutes and up to 120 minutes after inhalation of THC.	The study tested only single dose effects and not effects over the long term in a small sample size. Only two different low doses (0.5mg, 1.0mg) of the drug were tried. A single type of cannabis drug, one that was high in THC and low in CBD was included in the trial.
Chaves et al. 2020 [16]	Assessment of symptoms and quality of life using FIQ scores	Significant reduction on FIQ scores; notable increase in energy levels for daily activities	Size of the study population; short intervention period; no inclusion of washout period

Discussion

A host of studies have revealed several limitations with the current standard drug regimen being prescribed to patients suffering from fibromyalgia syndrome and for the management of neuropathic pain with regard to their inadequate pain relief as well as abuse potential and compliance issues. Cannabinoids have emerged as potential natural alternatives to the current regimens [17]. However, there is a dearth of data surrounding the use of cannabinoids as pain management alternatives, partially because cannabis was earlier banned for many years in some countries and is still considered a social taboo. The lack of adequate clinical evidence further hinders its medical prescription [18]. Despite the limited information available, the findings surrounding the use of cannabis and its beneficial effects to relieve the main symptoms of neuropathies and fibromyalgia are promising.

Weizman et al. (2018) conducted a double-blind crossover RCT to study the effect of THC through the sublingual route on neuropathic pain while also observing the accompanying changes in the neural response [12]. The trial included 15 male individuals having had medium to high lumbar radicular neuropathic pain for at least the past six months. The participants received a dosage of 0.2mg/kg of THC oil in the intervention group and a placebo oil in the control group, both sublingually. The pain was measured using the VAS scale both before and after the intervention. The test and control groups were interchanged a week later to repeat the experiment. The difference in the pain score was 8.7 for the placebo and 18.8 for the test group [12]. The results showed a staggering reduction in pain and an associated decrease in the anterior cingulate cortex (ACC) activity after cannabis administration, as compared to the placebo. As Xiao et al. (2021) had demonstrated that increased connectivity in the ACC was associated with painful states, the change in the ACC response with THC in this study further corroborated the VAS statistics obtained from the trial [19]. Hence, through this study, it has been demonstrated that the cannabis-derived product indeed played a role in decreasing neuropathic pain [12].

A similar study carried out by Weizman et al. (2024) further validated the positive effect of cannabinoids on chronic pain management [14]. The study was a double-blind crossover RCT that included twelve male participants experiencing neuropathic pain in the lower limbs for more than six months. The test group received a dosage of 0.2mg/kg sublingual THC oil and the control group received hemp oil via the same route. The effect on the autonomic system and conditioned pain modulation (CPM) was observed after the drug intervention. Consequently, increased parasympathetic activity and an improved CPM were noted, which were both indicative of a decrease in chronic pain [14]. This finding was supported by the findings of Hohenschurz-Schmidt et al. while experiencing chronic pain, there is an associated increased activity in the ACC [20]. The studies carried out by Weizman et al. (2018) and Weizman et al. (2024) have had their study outcomes in favor of the chronic pain-relieving potential of cannabinoids while a major drawback of both was that they had solely included male participants [12,14]. 

Another study that was in line with the beneficial effects of cannabis was carried out by Almog et al. (2020) [15]. It was a double-blind crossover RCT including both male (70%) and female participants suffering from chronic pain which was measured to be ≥ 6 on the VAS and the effect on the pain status was assessed along with the efficacy of the mode of drug delivery. This study further bolstered the medical potential of cannabinoids as THC had proved to decrease the pain intensity more than the placebo, with both its doses of 0.5mg and 1.0mg delivered via the Syqe inhaler. The decrease in the pain measured by VAS between baseline and post-intervention was a staggering 39.4% with 1.0mg THC and merely 12.7% with the placebo. The study has substantiated that THC is effective in decreasing neuropathic pain in males as well as females. It has also demonstrated that even a small dose of cannabinoids had the capacity to effectively reducing the pain over a short term when delivered through an inhaler along with strikingly minimal (95% mild) side effects. This study has proven that cannabinoids are efficacious as pain-relieving drugs in more than one form, making them a potential asset in chronic pain management. This point was further supported by a double-blind crossover RCT conducted by Xu et al. (2020) which assessed the efficacy of topical cannabidiol oil [13]. The trial included both males (62.1%) and females (37.9%) experiencing symptomatic peripheral neuropathy for at least three months. The test group was given each a topical cream containing 250 mg of CBD per 3 fl. oz container which had to be applied four times daily over four weeks while the control group applied placebo emu oil. The pain intensity of each group was then assessed via the NPS. A remarkable decrease was noted in sharp pain, intense pain as well as itchy sensation as compared to the placebo. Another important finding was that no side effects were recorded in the four-week neuropathic pain management trial with topical cannabinoid. Xu et al. went further to claim that transdermally delivered cannabinoids would be a more efficacious way of managing this condition. This claim was congruent with a statement made by Pergolizzi et al. (2018) mentioning that cannabinoids were the best analgesic option for neuropathic pain [21].

A trial led by Chaves et al. (2020) studied the effect of cannabis oil on 17 females suffering from fibromyalgia [16]. The study was a double-blind placebo-controlled RCT and the effect of the intervention was assessed via the Fibromyalgia Impact Questionnaire (FIQ). The participants were only females. The test group received on average 3.6 drops/day of THC-rich cannabis oil while the placebo group received an average of 4.3 drops/day olive oil. The study outcome showed a decrease in the FIQ mean score of 9 with the placebo and a notable decrease of 45 with the cannabinoid. Through the FQI, it was evident that the cannabinoids had impressively enhanced the well-being and quality of life of the participants over the eight weeks. This outcome was consistent with the findings of Hohenschurz-Schmidt et al. which had shown that fibromyalgia was associated with a heightened low frequency to high frequency ratio (LF/HF) and that of Weizman et al. (2024) which had demonstrated that THC was responsible for a decrease in LF/HF ratio, an increased parasympathetic activity and therefore a lowered pain sensation [14,20]. All the studies conducted have led to the idea that cannabinoids are indeed potential alternatives for pain management in both neuropathic pain and fibromyalgia. These drugs were effective analgesics as compared to placebos in different doses as well as various modes of delivery such as sublingual and topical.

The positive effects of cannabinoids in pain management are clear and their merit in the treatment thereof is evident; furthermore, the fact that cannabinoids are natural garners it support over traditional synthetic and semi-synthetic drugs. A limitation to both the further studying and implementation of the drug is the fact that social taboos and outdated legislation still exist and is likely retarding the propulsion of cannabinoids onto the global pharmaceutical market. It is likely that legislation will catch up with these developments and findings which will ultimately lead to an increase of research and ultimate acceptance of cannabinoids into the traditional pharmaceutical market as an equal to their conventional synthetic competitors.

## Conclusions

Cannabis-derived medications are novel in the pharmaceutical industry, with insufficient evidence available on their long-term adverse effects and drug interactions, due to their ambiguous legal status and heightened social taboos globally. In order to make cannabinoids a more acceptable alternative for chronic pain management, further trials are required to gather evidence with a larger sample size and a more diverse population. The potential benefit of cannabinoids is that they are naturally derived drugs that have already been shown to have the potential to effectively decrease chronic pain with minimal side effects as compared to the standard drugs being used. The ability of cannabinoids to provide pain relief with minimal side effects and concurrently be a naturally derived product may potentially be a life-changing alternative that the pharmaceutical market is in dire need of.
